# A Successful Percutaneous Endoscopic Gastrostomy Tube Feeding over Two Decades with No Complication: A Rare Case Report

**DOI:** 10.7759/cureus.5340

**Published:** 2019-08-07

**Authors:** Amrendra Mandal, Paritosh Kafle, Jasdeep S Sidhu, Muhammad Hassan, Vijay Gayam

**Affiliations:** 1 Internal Medicine, Interfaith Medical Center, Brooklyn, USA

**Keywords:** percutaneous endoscopic gastrostomy, mechanical dysphagia, enteral tube feeding

## Abstract

Percutaneous endoscopic gastrostomy (PEG) feeding is a common and widely performed procedure appropriate for long-term enteral nutrition in patients with multiple indications. We present the case of a 59-year-old woman with a PEG tube placed owing to complication following thyroid surgery approximately 20 years ago, representing the most extended duration of PEG tube feeding without any significant complication for chronic mechanical dysphagia. This case highlights the importance of PEG feeding, where this route can be used indefinitely in an appropriate clinical setting without complications. Interestingly, self-replacement of PEG tube was performed by the patient herself whenever she noticed clogging up of tube while self-feeding.

## Introduction

Percutaneous endoscopic gastrostomy (PEG) tubes are placed for many conditions wherein a patient is unable to intake food orally. The PEG tube was first used in 1980, where an endoscope was used to place a feeding tube into a patient’s stomach [1]. PEG tube use provides more natural nutrition than parenteral feeding and is usually a safe procedure. The most important role of this tube is to provide a route for enteral feeding and hydration and to administer medication in patients who are a potential candidate for inadequate or absent oral intake. PEG feeding is not recommended for short-term use because the 30-day mortality after PEG placement is substantial [1,2]. A gastrostomy tube placed endoscopically is usually a better choice than surgical placement [3,4]. We describe a case of a patient using PEG tube feeding for almost 20 years with no complications.

## Case presentation

**Figure 1:**
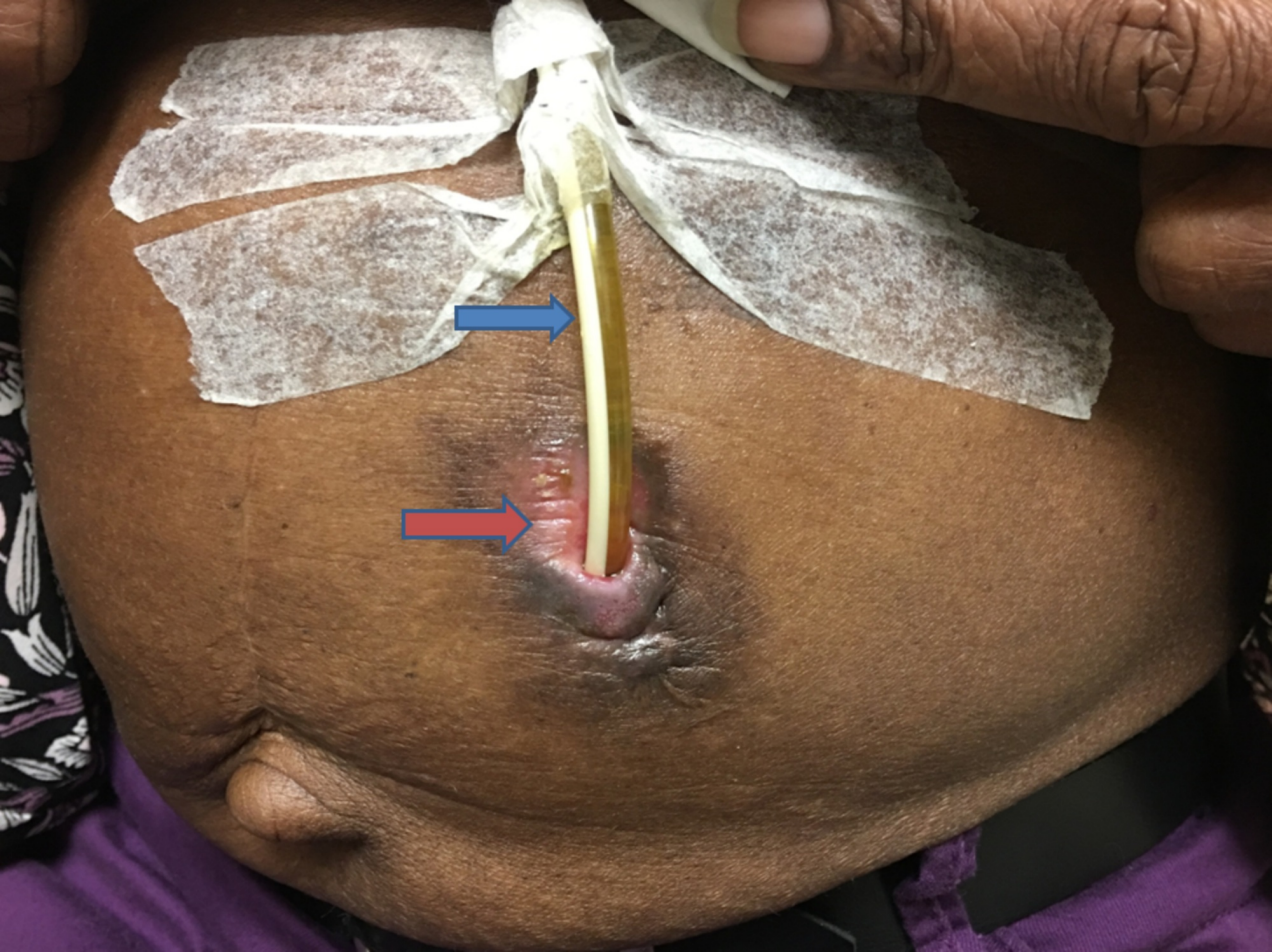
Image of the abdomen showed replacement PEG tube placed in situ at the left upper quadrant. Blue arrow indicates PEG tube and red arrow indicates the clean and uninfected peristomal area. Abbreviations: PEG, percutaneous endoscopic gastrostomy.

 A 59-year-old woman with a past medical history of chronic dysphagia following PEG tube replacement due to thyroid surgery complications approximately 20 years ago presented to the clinic for the establishment of primary care and evaluation for chronic dysphagia as she wished to eat food naturally. She recently moved from Jamaica, where the PEG tube was initially placed soon after the thyroid surgery. Since then, she reported that she has been on PEG tube feeding due to the inability to resume oral feeding. Interestingly, she reported replacing the PEG tube herself through the original track whenever she experienced a tube clog. She reported chewing her food, then pushing it through the tube with a syringe. On examination, we noted a well-healed scar at the base of her neck. The PEG tube was located at the left upper abdomen with no signs of inflammation or drainage at the peristomal area (Figure 1).

**Figure 2:**
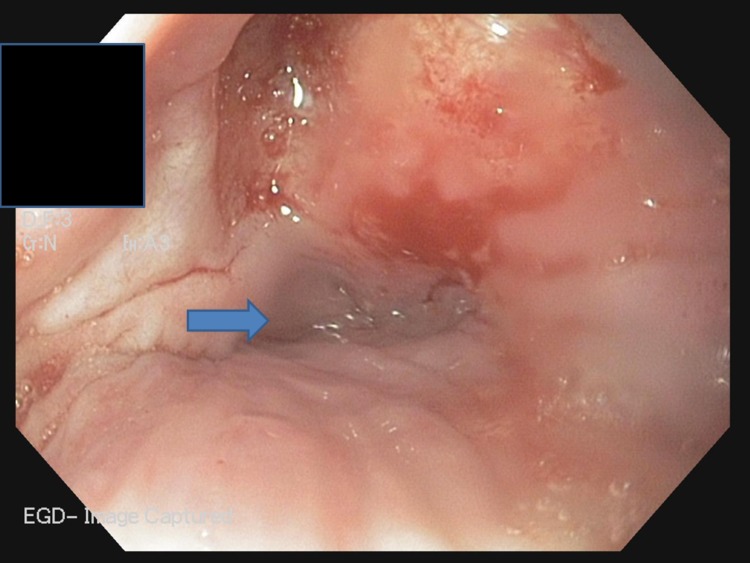
EGD showing scar present in the hypopharynx as well as single diverticulum (blue arrow) in the cricopharyngeus without further advancement of the scope. Abbreviations: EGD, esophagogastroduodenoscopy.

We also noted a laparotomy scar from a previous surgery for intestinal obstruction. The other physical examination findings were not remarkable. Her laboratory evaluations including complete blood count, chemistry panel, and lipid panel with normal findings; the viral serology for Hepatitis B and C were negative. Ultrasonography of the abdomen revealed no significant findings. Recent esophagogastroduodenoscopy revealed scarring in the hypopharynx as well as single diverticulum in the cricopharyngeus without further advancement of the scope (Figure 2).

Subsequently, a diatrizoate swallow study revealed the inability of the passage of contrast out of hypopharynx to the esophagus. She was ultimately counseled to depend on PEG feeding as she was maintaining her nutritional status with PEG feeding, and there were no better alternatives considering the potential worse consequences of surgery.

## Discussion

PEG feeding plays a significant role in the management of patients with poor voluntary oral intake, mechanical dysphagia, or neurological causes [5]. The two main goals for PEG placement are a viable route for feeding access and gastric decompression [6]. The patient should have normal or near-normal gastric and small bowel motility, which are vital prerequisites for PEG tube placement [7]. The beneficial effects of gastrostomy feeding on morbidity and mortality have been described only in specific subgroups of patients [8,9]. The average lifespan of PEG tubes has been reported to be one to two years and is based on tube degradation [10].

PEG tubes use has been increasing, especially for situations where long-term outcomes are uncertain. Few studies show that while short-term survival rates following PEG tube placement are high (80% to 90%), long-term survival rates are low [2]. Short-term mortality is commonly attributed to the co-morbidities rather than to the PEG tube itself [11]. The high long-term mortality rates following the placement of a gastrostomy tube are mostly from severe co-morbidities of the patients in whom tubes are most commonly placed. A US study of mortality rates following PEG tube placement in more than 180,000 patients showed an in-hospital mortality rate of 11% [12]. There are several factors related to the high risk of death, including advanced patient age, and co-morbidities such as heart failure and renal failure. However, female gender, diabetes mellitus, and paralysis are associated with lower mortality rates.

Several studies report that long-term survival rates are low (approximately 40% at 12 to 18 months, and 20% at three years) [3,8,13]. In the largest study, involving 80,000 patients with gastrostomy tubes, the majority of patients (75%) were older than age 75 [14]. The most common indications for tube placement were cerebrovascular disease, tumors, fluid and electrolyte disorders, and aspiration pneumonia. Survival rates at one year and three years were 37% and 19%, respectively, and the overall in-hospital mortality rate was 15%.

Approximately 13% to 40% of patients with PEG placement experience minor complications such as maceration due to leakage of gastric contents [3,15]. Serious complications have been reported in 0.4% to 4.4% of procedures. In one study, overall mortality from PEG tube placement was below 1% with minor complications occurring in 17% to 24% of patients; major complications requiring surgical intervention occurred in only 6% to 7% [16].

## Conclusions

PEG tube placement has developed into a standard procedure to secure gastric access. Gastrostomy tubes can be placed endoscopically, surgically, and radiologically. There is significant variation in survival after PEG tube insertion. The vulnerable groups are elderly patients with co-morbid illnesses. Despite reports of high short-term survival and low long-term survival, this case presents evidence that PEG tube placement is not only safe but may be used for an indefinite period in patients with mechanical dysphagia without critical illness.

## References

[1] Kobayashi K, Cooper GS, Chak A, Sivak Jr MV, Wong RC (2002). A prospective evaluation of outcome in patients referred for PEG placement. Gastrointest Endosc.

[2] Grant JP (1988). Comparison of percutaneous endoscopic gastrostomy with Stamm gastrostomy. Ann Surg.

[3] Ho C-S, Yee AC, McPherson R Complications of surgical and percutaneous nonendoscopic gastrostomy: review of 233 patients. Gastroenterology.

[4] Oh DJ, Kim B, Lee JK (2016). Can percutaneous endoscopic gastrostomy be carried out safely in the elderly?. Geriatr Gerontol Int.

[5] Blumenstein I, Shastri YM, Stein J (2014). Gastroenteric tube feeding: techniques, problems and solutions. World J Gastroenterol.

[6] McClave SA, Ritchie CS (2006). The role of endoscopically placed feeding or decompression tubes. Gastroenterol Clin North Am.

[7] Itkin M, DeLegge MH, Fang JC (2011). Multidisciplinary practical guidelines for gastrointestinal access for enteral nutrition and decompression from the Society of Interventional Radiology and American Gastroenterological Association (AGA) Institute, with endorsement by Canadian Interventional Radiological Association (CIRA) and Cardiovascular and Interventional Radiological Society of Europe (CIRSE).. Gastroenterology.

[8] Taylor CA, Larson DE, Ballard DJ, Bergstrom LR, Silverstein MD, Zinsmeister AR, DiMagno EP (1992). Predictors of outcome after percutaneous endoscopic gastrostomy: a community-based study. Mayo Clin Proc.

[9] Finocchiaro C, Galletti R, Rovera G, Ferrari A, Todros L, Vuolo A, Balzola F (1997). Percutaneous endoscopic gastrostomy: a long-term follow-up. Nutrition.

[10] ASGE Technology Committee, Kwon RS, Banerjee S, Desilets D (2010). Enteral nutrition access devices. Gastrointest Endosc.

[11] Fisman DN, Levy AR, Gifford DR, Tamblyn R (1999). Survival after percutaneous endoscopic gastrostomy among older residents of Quebec. J Am Geriatr Soc.

[12] Arora G, Rockey D, Gupta S (2013). High in-hospital mortality after percutaneous endoscopic gastrostomy: results of a nationwide population-based study. Clin Gastroenterol Hepatol.

[13] Callahan CM, Haag KM, Weinberger M, Tierney WM, Buchanan NN, Stump TE, Nisi R (2000). Outcomes of percutaneous endoscopic gastrostomy among older adults in a community setting. J Am Geriatr Soc.

[14] Grant MD, Rudberg MA, Brody JA (1998). Gastrostomy placement and mortality among hospitalized Medicare beneficiaries. JAMA.

[15] Hull M, Rawlings J, Field J (1993). Audit of the outcome of long-term enteral nutrition by percutaneous endoscopic gastrostomy. Lancet.

[16] Karhadkar AS, Schwartz HJ, Dutta SK (2006). Jejunocutaneous fistula is manifesting as chronic diarrhea after PEG tube replacement. J Clin Gastroenterol.

